# Pediatric necrobiosis lipoidica: Adults and kids are not the same

**DOI:** 10.1016/j.jdcr.2026.04.073

**Published:** 2026-05-25

**Authors:** Sophie-Anne Savard, Juliette Charbonneau, Elen Grigorchuk, Afshin Hatami, Danielle Marcoux, Catherine McCuaig, Isabelle Auger, Julie Powell, Jerome Coulombe

**Affiliations:** a Division of Dermatology, Department of Medicine, CHUM, Universite de Montreal, Montreal, Canada; b Faculty of Medicine, School of Medicine, Universite de Montreal, Montreal, Canada; c Division of Dermatology, Department of Pediatrics, CHU Sainte-Justine, Universite de Montreal, Montreal, Canada; d Division of Dermatology, Department of Pediatrics, CHUL, Universite Laval, Quebec City, Canada

**Keywords:** clinical cases, diabetes, medical dermatology, panniculitis, pediatrics, ulcers

Necrobiosis lipoidica (NL) is a rare cutaneous granulomatous disorder of unknown etiology.^1^ In adults, it has been variably associated with diabetes mellitus (DM).^1^ NL is less well described in pediatric patients due to its rarity.^2^ We conducted a descriptive retrospective chart review of NL pediatric cases from 1980 to 2025 at CHU Sainte-Justine, Montreal, Canada. Patients were included who were younger than 18 years of age at the time NL was diagnosed clinically or by histology and supported by photography. Descriptive analyses were used. In our cohort of 10 children with NL (Table I), 8 were girls, and 90% of patients had type 1 diabetes (T1DM). The mean age at diagnosis of DM was 4.5 years old, and 12.7 years for NL. Eight had a poor control of their DM with a mean HbA1c at 10.3%. Seven patients had classic pretibial plaques (Fig 1). All our patients received a variety of topical treatments as first line therapy (high-potency corticosteroids, calcineurin inhibitors, lactic acid, retinoid, or JAK-inhibitors). Topicals conferred improved control of symptoms with limited improvement in appearance. Four patients agreed to intralesional corticosteroid injections (triamcinolone acetonide 3-10 mg/mL) that provided partial improvement on disease activity, meaning a slight decrease in reported symptoms and the absence of enlargement of the skin lesions or avoiding progression to ulceration and/or induration. The mean duration of lesions was 3.8 years, with 90% of patients having ongoing NL activity at the time of last follow-up. Congruent with the limited literature in children,^2^ our patients with NL were predominantly teenage girls, had poorly controlled T1DM, and partial response to medical interventions. In contrast with NL in adults,^1^ pediatric NL incidence is lower, but more frequently associated with DM.^2^ Few diabetic end-organ damage were present in our adolescent patients with poorer glycemic control than in adult NL fueling the hypothesis that NL development might be more related to hyperglycemia than a microangiopathic pathophysiology process.^3^ There is no validated therapeutic management for pediatric NL.^2^ In our study, treatment responses were unsatisfactory with best results achieved through painful and unpopular intralesional corticosteroids. Many therapeutic options including phototherapy, immunosuppressants, and biologics have been described, again with limited success.^2^ Therapeutic breakthrough might be on the horizon with the use of topical and oral JAK inhibitors for classic and ulcerated NL respectively.^4^ Efficient, pain-free and safe drugs are always welcome for pediatric patients giving the NL treatment failure rate and long-standing activity of the disease into adulthood. Skin lesions with poorly controlled ulcerations have been recently associated with the development of squamous cell carcinomas in NL adult patients.^5^ The median time to skin cancer growth was 6 years.^5^ Only one of our patients had recurrent ulcerations. However, given the chronicity of NL and the risk of neoplasia in ulcerated NL, better treatments are needed. The limitations of our study include the small number of patients lacking statistical significance, retrospective design, loss of patient’s to follow-up at 18 years of age and a single medical center only. Nonetheless, pediatric NL differs from adult NL with its higher rate of DM association, poorer glycemic control in teenagers, and limitations in therapeutic option choice. Further studies of necrobiosis lipoidica in children are advisable.

**Table I:** Characteristics of pediatric patients with necrobiosis lipoidica (NL)

Patient number	Patients’ characteristics				DM control		Characteristics of NL lesions				Prognostic with treatments	
	Sex	Age at NL diagnosis (y)	Duration of NL (y)	Age at DM diagnosis (y)	HbA1c (mmol/mol)	Duration of DM (y)	Medical comorbidities	Lesions count	Lesions morphology	Lesions distribution	Treatment	Response
1	F	12	9	2	0.097	21	None	Multiple	Y	Pretibial	T + I + S	P
2	F	16	3	8	0.129	23	DKA Nephropathy	Three	Y	Pretibial	T + Pulse-dye laser	P
3	F	14	1	NA	0.055	1	None	Two	N	Thigh	Weight loss + T	D
4	M	16	1	4	0.130	1	DKA Plaque psoriasis	Solitary	Y	Pretibial	T + I	P
5	F	13	1	5	0.089	1	DKA	Solitary	Y	Hand	T	P
6	F	11	7	1	0.080	35	DKA Localized alopecia	Multiple	Y Preceded by multiple GA	Pretibial	T + I + S	P
7	F	13	5	13	0.155	45	DKA Nephropathy Retinopathy Thyroiditis	Multiple	Y Frequent ulcerations	Pretibial	T + S	P
8	F	14	4	NA	Normal	4	None	Three	Y	Pretibial	T + I	P
9	M	4	1	2	0.095	1	DKA	Solitary	Y	Bicep	T	P
10	F	14	10	1	0.100	10	DKA	Multiple	Y	Pretibial	T	P

*D*, Definitive improvement; *DKA*, diabetic ketoacidosis; *DM*, diabetes mellitus; *F*, female; *GA*, granuloma annulare; *I*, intralesional steroids; *M*, male; *N*, panniculitis nodules; *NA*, not available; *NL*, necrobiosis lipoidica; *P*, persistence of lesions at last visit; *S*, systemic drugs, consisting of aspirin and pentoxifylline; *T*, topical drugs, including topical corticosteroids [mid- to potent to very potent], with or without vitamin D analogues, calcineurin inhibitors, retinoids, lactic acid, and JAK inhibitors [1 and 2]; *Y*, yellowish telangiectatic atrophic plaques.

**Fig 1:**
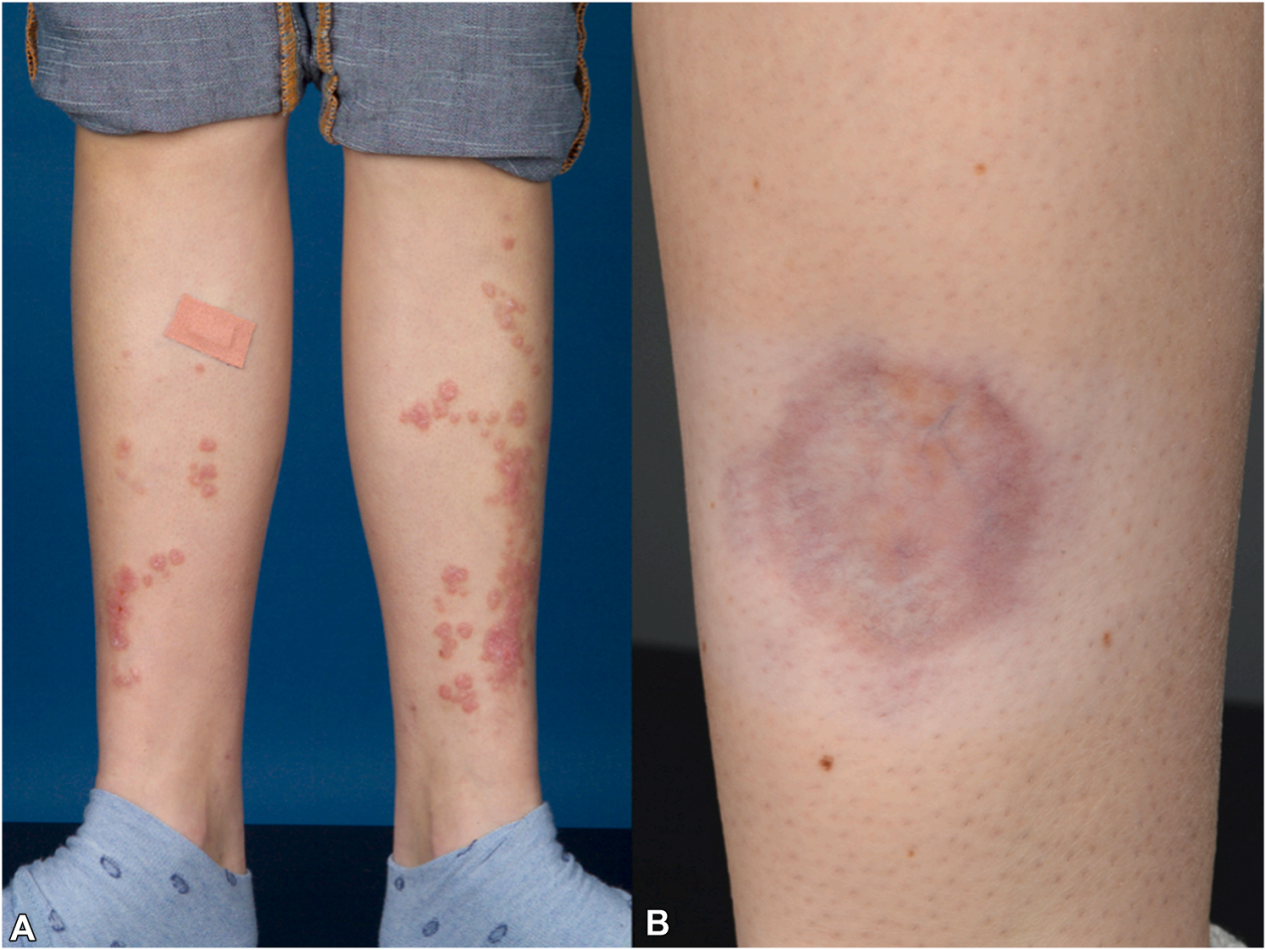
**A** and **B,** Necrobiosis lipoidica (NL) in a pretibial distribution presenting as multiple yellowish telangiectatic atrophic plaques in 2 pediatric patients form our cohort.

### Declaration of generative AI and AI-assisted technologies in the writing process

AI was not used.

## Conflicts of interest

None disclosed.

## References

[1] Hashemi D.A., Brown-Joel Z.O., Tkachenko E. (2019). Clinical features and comorbidities of patients with necrobiosis lipoidica with or without diabetes. JAMA Dermatol.

[2] Schiefer-Niederkorn A., Sadoghi B., Binder B. (2023). Necrobiosis lipoidica in childhood: a review of literature with emphasis on therapy. J Dtsch Dermatol Ges.

[3] Hammer E., Llienthal E., Hofer S. (2017). Risk factors for necrobiosis lipoidica in type 1 diabetes mellitus. Diabet Med.

[4] Hughes A.N., Li X., Lehman J.S. (2024). Drug repurposing using molecular network analysis identifies Jak as targetable driver in necrobiosis lipoidica. JID Innov.

[5] Harvey J.A., Severson K.J., Brumfiel C.M. (2022). Necrobiosis lipoidica-associated cutaneous malignancy. J Am Acad Dermatol.

